# Correlation of the ALA-PDT Treatment Efficacy and the HPV Genotype Profile of Genital Warts after Cryotherapy Failure and Podophyllotoxin Therapy in Male Patients

**DOI:** 10.3390/life11020146

**Published:** 2021-02-14

**Authors:** Witold Owczarek, Monika Slowinska, Irena Walecka, Magdalena Ciazynska, Dorota Nowicka, Leszek Walczak, Elwira Paluchowska

**Affiliations:** 1 Department of Dermatology, Military Institute of Medicine, 01-755 Warsaw, Poland; witold.owczarek@dermedicus.pl (W.O.); lwalczak@wim.mil.pl (L.W.); epaluchowska@wim.mil.pl (E.P.); 2 Clinic of Dermatology, Centre of Postgraduate Medical Education, Central Clinical Hospital of the Ministry of the Interior, 02-097 Warsaw, Poland; irena.walecka@cskmswia.pl; 3 Department of Proliferative Diseases, Nicolaus Copernicus Multidisciplinary Centre for Oncology and Traumatology, 87-100 Torun, Poland; ciazynska.magdalena@gmail.com; 4 Department of Dermatology and Venereology, Medical University of Warsaw, 02-091 Warsaw, Poland; Dr.nowicka@wp.pl

**Keywords:** genital warts, ALA-PDT, DNA HPV, real-time PCR, treatment efficacy

## Abstract

Background: Genital warts are the manifestation of the human papillomavirus (HPV) infection, which may last for weeks or months before the clinical presentation. The primary aim of the study was the correlation of the DNA HPV genotypes eradication with the treatment response in male patients with persistent genital warts. Methods: Twenty-one male patients (age range: 22–58) after failure of cryotherapy and podophyllotoxin treatment were enrolled in the study. Genetic tests (Real Time - PCR method) analyzed the presence of DNA-HPV before and 6 months after four sessions (4 weeks apart) of photodynamic therapy with 5-aminolaevulinic acid (ALA-PDT). The treatment efficacy was evaluated before each PDT session and at the end of the study. Results: The single HPV DNA type was present in 15/21 of the patients (13/15 HPV6). The high-risk HPV types were found in 8/21 subjects, of which 6/8 had several types. Six months after four sessions of PDT, complete response was found in 16/21 (76.19%; *p* = 0.0007) of patients, and DNA HPV clearance was found in 66.67% (*p* = 0.03). The eradication rate differed among patients with primary low-risk and high-risk HPV types—76.92% (10/13; *p* = 0.0003) and 50% (4/8; *p* = 0.05) respectively. Conclusion: ALA-PDT is an effective treatment even after the failure of previous modalities. The persistence of clinical lesions and high oncological risk HPV types should be an indication for treatment prolongation.

## 1. Introduction

Genital warts are a common symptom of human papillomavirus (HPV) infection. They develop within weeks or months after the infection, but in most cases, the virus can be latent for months or years. Risk factors for HPV infections include early onset of sexual activity, multiple sexual partners, high-risk sexual practices, and poor hygiene [1,2,3]. HPV can be divided into low-risk and high-risk types according to their oncogenic potential. HPV low-risk types include 6, 11, 40, 42, 43, 44, 54, 61, 70, 72, and 81, and the high-risk types are 16, 18, 31, 33, 35, 39, 45, 51, 52, 56, 58, 59, 68, 73, and 82. Genital warts are commonly associated with HPV6 and HPV11. However, studies conducted in patients with clinical lesions have shown the possibility of additional infection with many other types of HPV [4,5,6,7]. Therapeutic approaches used in the treatment of genital warts can be divided into surgical ones such as cryotherapy, surgical excision, curettage, electrocoagulation or ablative laser treatment, and pharmacological methods, which include local immune modulators such as 5% imiquimod cream or antimitotic agents such as the drug podophyllotoxin [1,8,9]. All the treatment methods currently available are associated with a high risk of recurrence. Therefore, the treatment of recurring HPV infections remains of key importance. Photodynamic therapy with 5-aminolaevulinic acid (ALA-PDT) as a treatment method for genital warts is becoming an increasingly common practice [10,11,12,13,14]. Studies have shown high efficacy of ALA-PDT both in the elimination of HPV infections and in the prevention of relapses.

The primary aim of the study was the correlation of the DNA HPV genotypes eradication with the treatment response in vaccine-naive patients. The secondary aim was the assessment of PDT treatment efficacy of persistent genital warts in adult male patients.

## 2. Material and Methods

The method of analysis was chosen from the prospective single arm observational study. The study was approved by the Ethical committee of Military Institute of Medicine (Number 100/WIM/2018) and was conducted in accordance with Declaration of Helsinki. The study was conducted in Dermatology Department of the Military Institute of Medicine in Warsaw.

As eligible for enrollment, we invited male patients >/= 18 years old who were initially diagnosed by a dermatology specialist with genital warts and underwent two unsuccessful therapies with cryotherapy (at least 2 sessions) and podophyllotoxin (used twice daily for 3 consecutive days repeated for up to 5 weeks). Before enrollment, each patient had to sign the Informed Consent approved by the Ethical Committee.

The exclusion criteria regarded the contraindications to ALA-PDT procedure.

At the baseline visit, the clinical examination was performed by a dermatology specialist and genital swabs used for HPV DNA testing were collected from all the lesional skin areas. The sterile polyester swabs were individually packaged and a specimen collection vial contained 1 ml of specimen transport media. The swabs were taken with a brush that was rubbed gently but firmly several times on the genital warts. After collection, the swab was placed in the sterile transport media vial; then, the end of swab was cut of to permit capping. The closed vial was kept at 4 °C and shipped to Synevo/Nucleagena laboratory on dry ice on the collection day. The swabs were repeated after 6 months follow-up after the 4th PDT session on primary areas of lesions’ manifestation and from new areas in case of the wide spreading of the genital warts.

The Laboratory Synevo/Nucleagena is regularly certified by INSTAND and QCMD and guaranteed that all real-time PCR procedures were performed in accordance with manufacturer’s instructions. The test detected and identified the 37 most common HPV genotypes (12 high-oncogenic types (strains numbered 16, 18, 31, 33, 35, 39, 51, 52, 56, 58, 59, and 67) and 25 low oncogenic types (among them the most common is 6, 11)) as well detected the co-existence of several types of the virus, e.g., low and high oncogenic.

**Figure 1:**
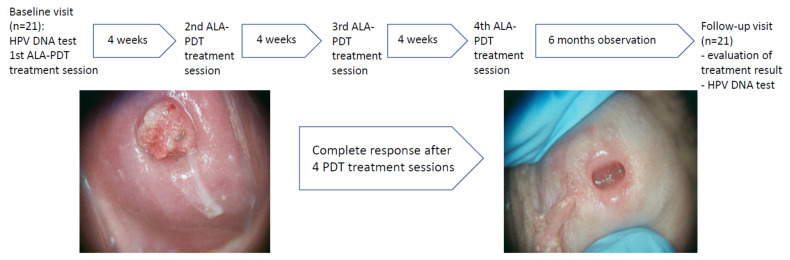
The study design with example of the clinical presentation of primary and final examination of the glans penis.

Starting from the baseline visit, 4 sessions of ALA-PDT were performed in 4-week intervals in each patient. Before each irradiation, 18% delta-aminolevulinic acid (5ALA) ointment freshly prepared by the hospital pharmacy was applied for three consecutive hours. Irradiation was performed with Waldmann PDT 1200L lamp with energy density of 100J/cm^2^ and 100Hz. Clinical evaluation was performed before each of the four ALA-PDT procedures as well as 4 weeks and 6 months after the last exposure. The clinical outcomes, adverse reactions, and recurrence rates were assessed. The photographic documentation was performed after receiving an agreement signed by the patient. Based on the photographic documentation performed on the baseline visit, the areas for the collection of the swabs after 6 months follow-up after the 4th PDT session were precisely specified (Figure 1).

All data were analyzed by Statistica 13.3. The numerical data were compared with the T-test, and the classified data were compared by the Chi-square test. *p* < 0.05 was considered statistically significant.

## 3. Results 

A total of 21 male patients fulfilled the inclusion criteria and were enrolled in the study. The median age was 30.0 years, the mean age was 34.7 years, and the age ranges were between 22 and 58 years. All patients presented genital warts in the penis area, and 7 participants additionally presented warts at the perianal area (Table 1). None of the patient had the intra-anal lesions. Based on anamnestic data, none of the 21 participants were previously immunized against HPV. The time interval between the first PDT session and the last introduced treatment (cryotherapy or podophyllotoxin) ranged between 1 and 6 months (mean 3.2 months). All patients presenting the genital warts located on the penis had lesions of diameter less than 5 mm, and 7/21 of patients had additionally lesions in the perianal area, some of which were 5–8 mm in size. The mean number of lesions was 9 (range 6–23).

**Figure 2:**
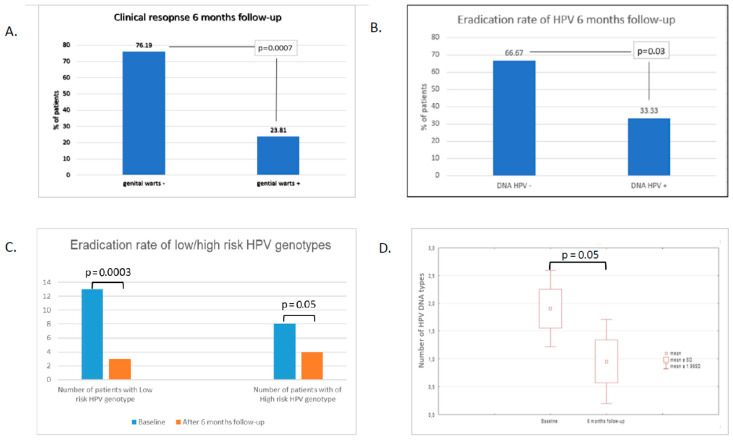
The results of treatment and DNA–HPV eradication rates before and 6 months after the four sessions of PDT treatment. Clinical examination of the presence of genital warts before and after 6-months follow-up showed statistically significant (*p* < 0.0007) differences (**A**), manifested also by the reduction in number of HPV lesions (**D**). The eradication rate of HPV was achieved in about 66% of patients (**B**) and was strongly correlated with the oncological potential of the HPV types (**C**).

The presence of HPV DNA was found at the baseline visit in all 21 subjects (Table 1). The single HPV DNA type was present in 15/21 of the patients. HPV DNA type 6 (13/15) was the most frequent in this group. Several types of DNA were detected in 6/21 of the patients. The presence of high-risk HPV types was found in 8/21 subjects, of which 6/8 had several types (Table 1). Six months after four sessions of PDT therapy, complete clinical response (complete response CR) was found in 76.19% (16/21) of patients, and in 5/21 (23.81%) there was clinical improvement with minimal genital warts (partial response). The difference was statistically significant (*p* = 0.0007) (Figure 2A).

HPV DNA 6 months after treatment was not found in 14/21 (66.67%), and in 7/21 (33.33%), there was no eradication of HPV DNA. The difference was statistically significant (*p* = 0.03) (Figure 2B). HPV DNA was found in all five patients with persistent genital warts (partial response) and in two patients without clinical lesions (complete response). The presence of high-risk HPV types after PDT was demonstrated in six out of the seven subjects. Of these, four had several types, and there was one HPV DNA type in two patients. In the group of five patients with genital warts, four had high-risk HPV types and one patient had low-risk HPV type 6. In patients with primary low-risk HPV type, 6-month HPV eradication was observed in 76.92% (10/13), and in the presence of high-risk HPV types, HPV eradication was found in 50% (4/8). The differences were statistically significant in the individual groups (Figure 2C).

The total number of virus genotypes found before treatment was 40 and decreased to 20. In 16/21 patients, the number of DNA HPV types decreased. In two patients, the number of types was found to have increased. The average number of HPV DNA types before treatment was 1.9 (min–max ± SD) (1–6 ± 1.6) and in the 6th month of observation, the average number of viruses was 0.95 (0–7 ± 1.77) (*p* = 0.05) (Figure 2D).

## 4. Discussion

The reported frequency of genital HPV DNA in men ranged from 1.3% to 72.9% (most studies report ≥20%), with the majority of HPV infections asymptomatic [2]. It is estimated that the probability of detecting a new HPV infection in men over a 12-month period is between 29% and 39% and does not significantly change across the lifespan [15,16]. The majority of HPV infections clear in less than 12 months; one study of men in the US reported a median time to clearance of 5.9 months, while a multinational study observed a median time to clearance of 7.5 months [15,16]. A multinational study reported that 50.5% of men were tested positive for at least one known HPV genotype, 25.7% were positive for multiple HPV genotypes, and 14.7% were positive for an unknown HPV type [17]. Among men with HPV infections 12.0% had oncogenic types only, 20.7% had non-oncogenic types only, and 17.8% had both oncogenic and non-oncogenic types [17]. The population of the patients in our study represents a specific group of men, who had persistent genital warts after two standard treatment methods, which could influence the results. Of these, 28.57% (6/21) of the patients were infected with multiple HPV genotypes and 38.09% (8/21) had the high-risk HPV genotypes, of whom 75% (6/8) had more than one oncogenic type. The presence of the HPV DNA 6 months after four PDT sessions was found in 33.33% (7/21) of patients (five patients had the partial response and two patients had the complete response) with the presence of high-risk HPV types in 85.7% (6/7) and multiple types in 57.14% (4/7) in this group. Our results stay in concordance with the results of previous publications [5,12,18,19,20,21]. Doorbar showed the persuasive evidence that HPV DNA can persist in the epithelium in the absence of a disease and the latent infections may persist for years or even decades [18]. Hu et al. have investigated the role of PDT in the treatment of subclinical or latent HPV infections with the conclusion that PDT treatment can effectively eliminate HPV, significantly reducing viral loads after three rounds of treatment (*p* < 0.001) [19]. Blomberg et al. indicated that the persistent HPV infection is the strongest risk factor for developing HPV-associated precancers and cancers [20]. The literature data indicate that patients with anogenital warts of one to six months duration were three times more likely to have oncogenic HPV infection compared to those with less than one month [7,21]. Hasanzadeh et al. have shown that the coexistence of high-risk and low-risk HPV types infection may be associated with both an increased risk of the development of genital warts and the risk of persistent and recurrent HPV infections [21]. Wang et al. showed that the total number of ALA-PDT sessions conducted in patients infected with multiple HPV types and/or high-risk HPV types was significantly higher compared to the group infected with only one type and/or with low-risk HPV types [12]. Those findings are confirmed by the results of our research, in which the presence of high-risk HPV types was associated with a worse clinical clearance rate and eradication rate of HPV (*p* = 0.05, Figure 2C) in comparison to the low-risk ones (*p* = 0.0003). Thus, the HPV DNA detection is the indicator of the infection severity and the prognostic factor of treatment duration [5,7,12]. Wang et al. made the duration of ALA-PDT treatment dependent on obtaining HPV DNA eradication confirmed by two negative tests [12]. Based on systematic reviews, the proper time point for the estimation of HPV DNA persistence after a follow-up of treatment is not precisely determined [22]. In our study, it was performed after 6 months after the last PDT session, similar to the studies of Wang et al. and Hu et al. [5,12]. In the case of female patients, it ranged between 1.5 and 80 months [22]. The most profound data regarding HPV DNA persistence, are available for female patients [22]. Median HPV persistence tended to decrease with increasing follow-up time, declining from 27% at 3 months after treatment to 21% at 6 months, 15% at 12 months, and 10% at 24 months [22]. On the other hand, the positive results of DNA HPV may represent either truly persistent infections, recurrent HPV infections, or newly acquired HPV infections in patients who are sexually active [22]. Hoffman et al. underlined those facts as of particular concern for studies with long time intervals (i.e., 12 and 24 months) between HPV tests [22]. The authors found that the HPV persistence estimates appeared to be higher in studies with a relatively shorter minimum interval between the first two testing time points to define persistence, suggesting that the clearance of HPV infections is what was actually observed in most cases [22].

Various treatment techniques are used to treat genital warts including ablation, immunotherapy, PDT, or a combination of all of the above [23,24,25]. The choice of therapeutic option should be individually adjusted for each patient based on clinical factors such as number, size, morphology, location, keratosis of warts, and infection’s course (new or recurrent) [5,8,23,24,25]. Patient preferences, such as the desire to be treated at home or in a clinic, and the convenience of the treatment regimen in terms of frequency and time, should also be considered. When planning treatment, the methods with the lowest risk of failure should be selected first [1,8,23,24,25]. However, the available treatment options are difficult to rank in terms of efficacy due to the lack of comparative head-to-head studies. The literature data indicate that more than 90% of cases of genital warts are caused by HPV type 6 [1,3,23,25]. This finding was also confirmed by 80.95% (17/21) of patients in our study. Cryotherapy is commonly used to remove warts in everyday practice. The clearance rate of cryotherapy used in cycles is estimated at 46–96% and the recurrence rate is estimated at 18–39% [3,8,25]. The main disadvantage of cryotherapy is the high recurrence rate and the need to repeat therapeutic interventions. Podophyllotoxin interrupts the division of infected cells, causing tissue necrosis [26]. The clearance rate of podophyllotoxin 0.15% cream or 0.5% alcoholic solution used in cycles amounts to 45–94% [3,8,25]. The recurrence rate of such treatment ranges from 8 to 100% [8,25]. The results indicate that ablative techniques are clinically more effective in the complete removal of genital warts, but they are characterized by a higher recurrence rate [3,8,25]. Photodynamic therapy is a modern therapeutic method, which can also be applied in the treatment of genital warts [10,11,12,13,14]. The principle of PDT is to induce phototoxic reactions through the synergy of photosensitizer and light. Zhang et al. using ALA-PDT once a week for 3 weeks and found that 95.27% of the patients had their lesions resolved [13]. The authors also showed that the efficacy of three PDT cycles was significantly higher compared to only one or two PDT cycles. Additionally, the clearance rate of lesions with a diameter of less than 5 mm was significantly higher than of larger ones. At 24 weeks after the end of ALA-PDT, the total recurrence rate was 16.20%. In the evaluation study of Du et al. comparing the effectiveness of photodynamic therapy with topically applied 5-aminolevulinic acid and CO_2_ laser for the treatment of cervical condylomata acuminate, the eradication rate of HPV infection after 3 months was 90.20% and 65.80%, respectively, but the CR rate in the group treated with ALA-PDT was also significantly lower (90.2%) than in the CO_2_ laser group (96.2%) [10]. Hu et al. demonstrated that ALA-PDT is a particularly suitable method for men with warts in the urethral fossa and achieved CR in 98.8% of men after fourfold treatment and 91.7% in women after fivefold treatment [11]. The authors pointed out that in three patients with no improvement obtained after fourfold treatment, the additional one PDT session was successful [11]. In our study, clinical complete response after fourfold treatment of male patients was observed in 76.19%, which is the lower frequency in comparison to the results of studies cited above even those conducted on the female population, which achieves worse outcomes than male patients [5,10,11,12,13,14]. We presume that the difference in the efficacy of our PDT treatment results from the characteristics of the patients who underwent two ineffective therapies, comparing to the therapeutically naïve populations of cited studies. The influence of the duration of disease and the HPV genotype profile was discussed above. The efficacy of the Wang et al. study was influenced by the prolongation of the PDT treatment (mean number ranged between 3.2 and 6.7 per patient) according to double-negative HPV-DNA tests [12] The different periods of follow-up, which ranged between 3 and 6 months in cited studies, could have also an impact on the observed treatment efficacy in comparison to our study [5,10,11,12,13,14]. 

Nevertheless, this study proved that ALA-PDT is the effective option for about 66% of male patients with persistent genital warts previously treated ineffectively. As the clinical complete response did not indicate the DNA HPV eradication, the objective response should be confirmed by the RT-PCR evaluation, which might influence the further decision about the prolongation or modification of treatment.

## 5. Conclusions

ALA-PDT may be an important method for the treatment of genital warts, especially after the failure of previous treatments. The presence of high-risk DNA HPV types may be associated with a weaker response to PDT and the indication for the therapy prolongation.

## Figures and Tables

**Table 1:** The summary of the results of the human papillomavirus (HPV) genotyping before and after 6 months follow-up combined with the results of photodynamic therapy (PDT) treatment. CR—complete response; PR—partial response;

Age	Site of Genital Warts	HPV Genotype before PDT Treatment	PDT Treatment Cycles	Response after 4 PDT Treatment Cycles	HPV Genotype Testing after 6 Months Follow-up
49	Penis	6	4	CR	Not detected
30	Penis, Perianal area	6,16,18,59	4	PR	6,16,18,59
27	Penis, Perianal area	6,31,39,45,51	4	CR	31,39,45
43	Penis	6	4	CR	Not detected
39	Penis	18,58	4	CR	Not detected
28	Penis	6	4	CR	Not detected
29	Penis	6	4	CR	Not detected
27	Penis, Perianal area	6	4	CR	Not detected
25	Penis	6	4	PR	6
32	Penis	51	4	CR	Not detected
58	Penis	6,16,51,52,56,59	4	PR	6,16,35,51,52,56,59
37	Penis, Perianal area	11,33,56,58	4	CR	Not detected
25	Penis	6	4	CR	Not detected
29	Penis, Perianal area	6	4	CR	Not detected
29	Penis, Perianal area	56	4	PR	56
22	Penis	6	4	CR	Not detected
30	Penis	6,11,31,58	4	CR	Not detected
28	Penis, Perianal area	6	4	PR	6,51
25	Penis	6	4	CR	Not detected
47	Penis	6	4	CR	16
39	Penis	6	4	CR	Not detected

## Data Availability

Data is contained within the article.
